# Advances in the Study of Metabolic Reprogramming in Gastric Cancer

**DOI:** 10.1002/cam4.70948

**Published:** 2025-05-14

**Authors:** Yu Rong, Yuanyin Teng, Xiaoying Zhou

**Affiliations:** ^1^ The First Clinical Medical College, Nanjing Medical University Nanjing China; ^2^ The Second Clinical Medical College, Nanjing Medical University Nanjing China; ^3^ Department of Gastroenterology The First Affiliated Hospital of Nanjing Medical University Nanjing China

**Keywords:** gastric cancer, metabolic reprogramming, metabolomics, molecular targets

## Abstract

**Background:**

Gastric cancer is one of the most prevalent malignancies of the digestive system and is associated with a poor prognosis, particularly in advanced metastatic stages, where the 5‐year survival rate is significantly low.

**Methods:**

Recent research has demonstrated that metabolic reprogramming—including alterations in glucose, lipid, and amino‐acid metabolism—plays a critical role in both the development and progression of this disease. To gain deeper insights into these metabolic shifts, scientists have increasingly employed metabolomics, a non‐invasive technique that detects and quantifies small molecules within cancerous tissues, thereby enhancing prognostic assessments.

**Aim:**

Analyzing the metabolic profiles of gastric‐cancer tissues can reveal significant changes in key metabolic pathways, which may open new avenues for targeted therapies and ultimately improve patient outcomes.

**Conclusion:**

This article reviews recent advancements in the study of metabolic reprogramming in gastric cancer, aiming to identify potential therapeutic targets and offer new hope to patients.

## 1 Introduction

As of 2020, gastric cancer ranks as the fifth most common cancer globally and is the fourth leading cause of cancer‐related deaths. Although it occurs worldwide, its prevalence is particularly high in regions such as East Asia and Eastern Europe [1]. Furthermore, The Cancer Genome Atlas (TCGA) analyzed 295 untreated gastric adenocarcinoma cases and classified them into four distinct subtypes: microsatellite instability‐high (MSI‐H) tumors, Epstein–Barr virus (EBV)‐positive tumors, chromosomal instability (CIN) tumors, and genomically stable tumors [2].

Multiple factors contribute to the development of gastric cancer, including race, gender, *Helicobacter pylori* infection, and genetic predispositions. Notably, *Helicobacter pylori* infection is the primary cause of noncardia gastric cancer. This bacterium, along with its virulence factors such as CagA and lipopolysaccharide (LPS), interacts with gastric epithelial cells, initiating chronic inflammation, mucosal damage, and both genomic and epigenetic alterations that ultimately lead to malignant transformation [3, 4]. Furthermore, Jennifer et al. [5] discovered that under iron‐deficient conditions, *H. pylori* infection enhances gastric cancer risk by altering bile acid metabolism, specifically by significantly increasing deoxycholic acid (DCA) levels. This finding emphasizes the importance of metabolic reprogramming in the progression of *H. pylori* ‐induced gastric cancer.

In contrast, cardia gastric cancer is linked not only to *Helicobacter pylori* infection but also to obesity and gastroesophageal reflux disease, exhibiting similarities with esophageal adenocarcinoma. This complexity highlights the urgent need to enhance our understanding of these diverse risk factors to formulate more effective prevention and treatment strategies [1, 2].

Treatment strategies for gastric cancer encompass surgical resection, radiochemotherapy, and immunotherapy. Surgical resection serves as the primary treatment for early‐stage disease and can significantly enhance the 5‐year survival rate, highlighting the critical importance of early diagnosis and timely intervention [6]. However, due to the often‐vague early symptoms, diagnosis is frequently postponed until the disease reaches advanced stages, resulting in poorer prognoses and elevated mortality rates [6]. Furthermore, recent advancements in immunotherapy and targeted biomarker therapies have expanded treatment options. Key molecular markers, such as PD‐L1 and CTLA‐4, are pivotal in these developments. Notably, the interaction between the programmed cell death 1 (PD‐1) receptor and its ligand PD‐L1 is among the most common and extensively studied mechanisms; this interaction inhibits the immune response of CD8^+^ cytotoxic T cells, thereby enabling tumor cells to evade immune surveillance [7].

Cancer immunotherapy seeks to leverage the immune system to eliminate cancer cells and regulate tumor growth. Immune checkpoint inhibitors (ICIs) function by obstructing the binding of CTLA‐4 and PD‐1 to their respective ligands, thereby facilitating a vigorous antitumor immune response and counteracting cancer‐induced immune evasion mechanisms [8]. To date, the Food and Drug Administration (FDA) has approved three ICIs that target different molecules for use in humans. The first ICI approved was ipilimumab, an antibody against CTLA‐4, which is utilized in the treatment of metastatic melanoma. Following this, a second class of ICIs was introduced—antibodies that inhibit the interaction between PD‐1, an inhibitory receptor on T cells, and its ligands PD‐L1 and PD‐L2—to avert T cell suppression [9, 10].

Recent studies have further elucidated the interplay between tumor metabolism and the immune response. For instance, Zhang et al. [11] demonstrated that major facilitator superfamily domain‐containing protein 2A (MFSD2A), a protein associated with lipid metabolism, suppresses TGFβ1 secretion by gastric cancer cells through the inhibition of COX2‐prostaglandin synthesis. This suppression activates CD8^+^ T cells and diminishes the population of immunosuppressive cells within the tumor microenvironment, thereby enhancing the efficacy of anti‐PD‐1 immunotherapy. In another study, glucose depletion in murine sarcoma cells was shown to inhibit mTOR activity, reduce glycolytic capacity, and decrease IFN‐γ production in T cells, collectively promoting tumor progression. Notably, blocking the PD‐1/PD‐L1 axis or CTLA‐4 reversed these effects, suggesting that immune checkpoint blockade may be particularly effective for patients with tumors exhibiting high glycolytic rates [12].

Metabolomic processes are closely linked to immunotherapy. Similar to PD‐L1, indoleamine 2,3‐dioxygenase 1 (IDO1) exhibits a potent immunosuppressive effect [7]. IDO1 is an intracellular heme‐containing metalloprotein that catalyzes the rate‐limiting step in the conversion of tryptophan to kynurenine (KYN) within the kynurenine pathway (KP) [7, 8]. Tryptophan depletion leads to apoptosis or dysfunction of CD4^+^ T cells, while the accumulation of kynurenine promotes the differentiation of regulatory T cells [7]. Moreover, IDO1 interacts with key immunological targets such as CTLA‐4 and PD‐1. For instance, CTLA‐4 expressed by regulatory T cells induces IDO1 expression in dendritic cells (DCs), and PD‐1/PTEN signaling is essential for maintaining Treg‐mediated immunosuppression in IDO1‐activated cells. Additionally, the expression of IDO1 in DCs is induced by the interaction of PD‐1 with PD‐L1 on mast cells and/or PD‐L2 on DCs [10]. The combination of IDO1 inhibitors with CTLA‐4 or PD‐1 inhibitors has been reported to enhance the efficacy of immunotherapy; for example, the combination of the first‐generation IDO1 inhibitor epalrestat with the PD‐1 inhibitor pembrolizumab has resulted in durable responses [8].

In summary, these therapeutic advancements offer new hope for gastric cancer patients, especially when early diagnosis is difficult. Targeted treatments focusing on specific biomarkers have the potential to significantly improve the prognosis of patients with advanced‐stage disease.

Metabolic reprogramming has long been recognized as a hallmark of malignant tumors [13]. It involves metabolic changes that enable cancer cells to secure the energy needed for survival and rapid proliferation even in challenging environments. One notable example is the Warburg effect [14], in which tumor cells preferentially utilize glycolysis over mitochondrial respiration for energy production despite the presence of adequate oxygen. Reflecting this phenomenon, gastric cancer exhibits alterations in several metabolic pathways, including those related to carbohydrates, lipids, and amino acids. In this review, we explore the metabolic mechanisms and signaling pathways that drive metabolic reprogramming in gastric cancer, aiming to clarify their roles in disease progression. Moreover, key proteins involved in these processes may serve as promising targets for novel therapies. Ultimately, a deeper understanding of these metabolic changes can illuminate the pathophysiology of gastric cancer and open new avenues for future treatment strategies.

## 2 Overview of Metabolic Reprogramming

During cancer progression, the metabolic demands of tumors continuously evolve. In the early stages, tumor growth relies primarily on nutrient uptake and biosynthesis. However, as the disease advances, tumors shift toward alternative metabolic pathways that help manage oxidative stress and enhance oxidative phosphorylation. This process, known as metabolic reprogramming, involves pathways frequently observed in rapidly growing tumors and cancer cells [15]. Metabolic reprogramming not only influences tumor initiation and expansion but also equips emerging tumor cells with the traits necessary for survival, immune evasion, and proliferation. Because early lesions often present subtle symptoms and metabolic abnormalities are difficult to detect [13], researchers have increasingly focused on changes within the tumor metabolic microenvironment (TME). A deeper understanding of these metabolic alterations is crucial for early detection and may reveal new therapeutic targets.

The TME encompasses the dynamic exchange and interaction of substances between cells and the extracellular matrix within tumor tissues. It consists of various cell types—including tumor cells, fibroblasts, immune cells, adipocytes, and endothelial cells [16]—that interact through direct contact and signaling pathways, engaging in both cooperative and competitive metabolic activities. Consequently, tumor growth depends not only on cancer cells themselves but also on the complex metabolic interactions among cancer cells, stromal cells, and even different cancer cell subpopulations—a phenomenon often referred to as “metabolic coupling” or “metabolic symbiosis” [17]. Recent research by Yang et al. [18] has further highlighted the critical role of exosomes in shaping the TME. Exosomes are small vesicles released by cells that carry bioactive substances such as proteins, RNA, transcription factors, and lipids.

Bioactive molecules can be transferred to neighboring cancer cells via exosomes, triggering metabolic changes that drive cancer progression. For instance, exosome‐mediated delivery of miR‐210 and miR‐155 prompts normal fibroblasts to boost anaerobic glycolysis while reducing oxidative phosphorylation, leading to metabolic reprogramming and extracellular acidification—processes that facilitate melanoma metastasis. Furthermore, the oncoprotein LMP1 transforms fibroblasts into cancer‐associated fibroblasts (CAFs), which secrete cytokines and chemokines (including IL‐6, IL‐8, CXCL10, and TGF‐β) to alter cancer cell metabolism. Notably, CAFs also modulate lipid metabolism in cancer cells, enhancing their migratory and invasive capabilities [19]. Collectively, these complex metabolic interactions underscore the critical role of the TME in cancer progression. In addition, exosomes regulate tumor‐associated macrophages, affecting tumor growth, metastasis, and therapy resistance. For example, Hilakivi‐Clarke et al. [17] reported that lactate produced by cancer cells creates an acidic environment that not only inhibits immune cell proliferation but also induces apoptosis in cytotoxic immune cells and shifts macrophages toward a phenotype that promotes cancer progression.

In gastric and colorectal cancer (CRC) cell lines, abnormal activation of KRAS upregulates the expression of the glucose transporter‐1 (GLUT1), enabling tumor cells to adapt to low‐glucose conditions and ensuring their survival [20]. Additionally, dysregulated fatty acid metabolism forces immunosuppressive cells to rely more on fatty acid oxidation (FAO), leading to immune dysfunction and altering immune phenotypes in a way that favors cancer growth [20]. Moreover, under nutrient stress—such as glucose or glutamine deficiency—tumor cells increase the expression of key enzymes for serine biosynthesis (e.g., PHGDH, PSAT1, and PSPH) to maintain redox balance and activate oncogenic pathways, including c‐Myc signaling [21]. Together, these metabolic adaptations enable tumor cells to thrive in harsh environments, highlighting the crucial role of metabolic regulation in tumor development.

Metabolic reprogramming is essential to cancer development and serves as a significant biomarker for both diagnosis and prognosis. Furthermore, targeting the metabolism of cancer cells presents a promising strategy for inhibiting tumor growth and metastasis. Thus, investigating metabolic reprogramming not only offers new tools for early diagnosis and prognostic evaluation but also lays the groundwork for innovative therapeutic approaches.

Table 1 provides a summary of the key aspects of metabolic reprogramming.

**TABLE 1:** Summary of metabolic reprogramming [1].

Topics	Summary
Metabolic reprogramming	Examines how tumors acquire nutrients, interact with their microenvironment, and promote growth. Discusses metabolic changes from exosomes, the role of lactic acid in immune suppression, tumor survival under stress, and the therapeutic potential of targeting metabolism
Tumor microenvironment	Examines tumor metabolism, emphasizing nutrient uptake, tissue interactions, metabolic coupling, and immune suppression
Metabolic coupling	Describes how tumor growth is enhanced by metabolic interactions between tumor cells and stromal cells, as well as among tumor cells with different metabolic profiles. This “metabolic coupling” involves nutrient and signal exchange to support mutual growth and survival
Exosome effects	Exosomes carry active substances that modify recipient cell metabolism, promoting cancer progression
Immune suppression	Lactic acid from tumors creates an acidic environment that suppresses immune cell growth, induces cell death, and shifts macrophages to cancer‐promoting types
Tumor cell regulation	Abnormal KRAS activation elevates GLUT1 levels, promoting metabolic adaptation and tumor cell survival in low‐glucose conditions
Applications	Metabolic reprogramming is a diagnostic biomarker for predicting cancer outcomes and a therapeutic target to inhibit tumor growth and spread

## 3 Metabolic Reprogramming and Gastric Cancer

### 3.1 Carbohydrate Metabolism in Gastric Cancer

Glucose is the primary energy source and an essential component for cell development. It fuels critical metabolic pathways—glycolysis, the citric acid cycle, and the pentose phosphate pathway—providing both energy and the molecular building blocks necessary for cellular functions. In oxygen‐rich tissues, pyruvate produced during glycolysis typically enters the citric acid cycle to generate energy and supply precursors for synthesizing fatty acids and amino acids [22, 23]. In contrast, cancer cells exhibit heightened glycolytic activity that leads to increased lactate production via lactate dehydrogenase (LDH) and reduced mitochondrial utilization of pyruvate. This metabolic shift, known as the Warburg effect or aerobic glycolysis, not only meets the elevated energy demands of cancer growth but also drives further metabolic reprogramming within tumors [24], underscoring the pivotal role of glucose metabolism in tumor biology.

**FIGURE 1:**
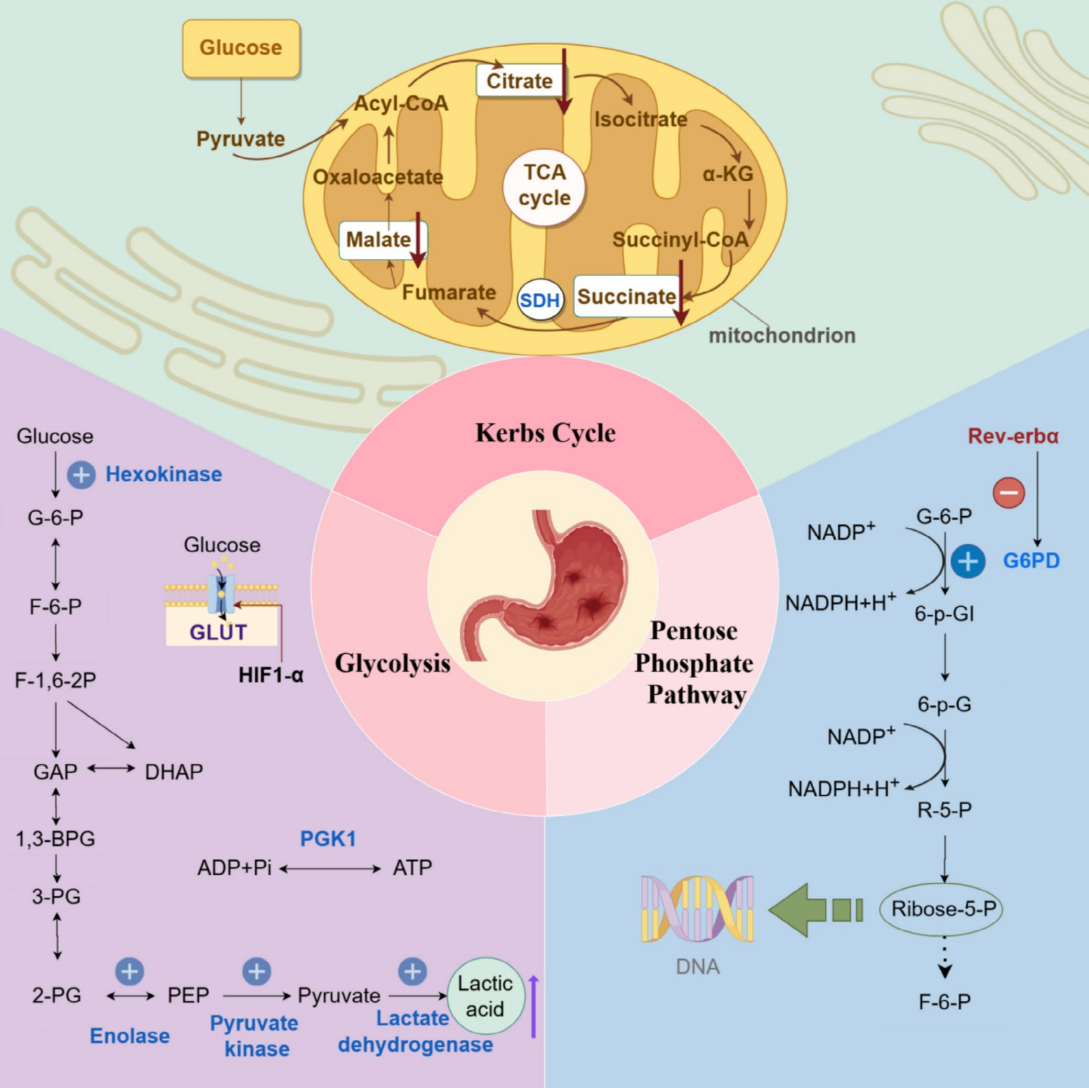
Mechanisms of carbohydrate metabolism in gastric cancer. Schematic representation of metabolic pathways in gastric cancer. The figure highlights key components of glucose metabolism, including glycolysis, the pentose phosphate pathway, and the tricarboxylic acid (TCA) cycle. Enzymes are shown in blue. Glycolysis, facilitated by hexokinase and phosphoglycerate kinase 1 (PGK1), produces pyruvate and lactate under hypoxic conditions. The pentose phosphate pathway, regulated by glucose‐6‐phosphate dehydrogenase (G6PD), generates ribose‐5‐phosphate for nucleotide synthesis. The TCA cycle within mitochondria highlights the role of succinate dehydrogenase (SDH) in energy production. Key molecular regulators, such as HIF1‐α and Rev‐erbα, significantly influence glucose metabolism and redox balance. 1,3‐BPG, 1,3‐Bisphosphoglycerate; 2‐PG, 2‐Phosphoglycerate; 3‐PG, 3‐Phosphoglycerate; 6‐p‐G, 6‐Phosphogluconate; 6‐p‐Gl, 6‐Phosphogluconolactonase; Acyl‐CoA, acyl‐coenzyme A; DHAP, dihydroxyacetone phosphate; F‐1,6‐2P, fructose‐1,6‐diphosphate; F‐6‐P, fructose‐6‐phosphate; G‐6‐P, glucose‐6‐phosphate; G6PD, glucose‐6‐phosphate dehydrogenase; GAP, glyceraldehyde‐3‐phosphate; GLUT, glucose transporters; HIF1‐α, hypoxia‐inducible factor 1‐alpha; PEP, phosphoenolpyruvate; PGK1, phosphoglycerate kinase 1; R‐5‐P, ribulose 5‐phosphate; Ribose‐5‐P, ribose 5‐phosphate; SDH, succinate dehydrogenase; Succinyl‐CoA, succinyl‐coenzyme A; TCA cycle, tricarboxylic acid cycle; α‐KG, alpha‐ketoglutarate.

Figure 1 illustrates the mechanisms of carbohydrate metabolism in gastric cancer.

#### 3.1.1 Glycolysis

Glycolysis is a fundamental pathway in carbohydrate metabolism that provides the energy necessary for growth and development. This process converts glucose into pyruvate through a series of enzyme‐driven reactions in the cytoplasm. Although glycolysis can occur without oxygen, it produces only a limited amount of energy. The pathway consists of 10 specific reactions, three of which are irreversible and catalyzed by key enzymes, including hexokinase (HK), phosphofructokinase (PFK), and pyruvate kinase (PK) [25]. Furthermore, studies have demonstrated that levels of several enzymes—such as HK, enolase (ENO1), PK, lactate dehydrogenase A (LDHA), glucose transporter (GLUT), and hypoxia‐inducible factor 1‐Alpha (HIF1‐α)—are significantly elevated in gastric cancer cells, underscoring their crucial role in glycolytic reprogramming.

##### 3.1.1.1 Hexokinase

HK, particularly the HK2 isoform, plays a crucial role in initiating glycolysis by converting glucose into glucose‐6‐phosphate. Although mammals express four hexokinase isoforms, overexpression of HK2 is notably linked to aggressive tumor features—including larger tumor size, lymph node metastasis, advanced cancer stages, and elevated alpha‐fetoprotein levels [26]. In support of its oncogenic role, Shao et al. [27] demonstrated that the stem cell factor SALL4 upregulates HK2 expression, thereby enhancing glycolysis and promoting cancer cell proliferation, migration, and invasion. Moreover, Wang et al. [28] demonstrated that HK2 modulates glycolysis in trastuzumab‐resistant HER2‐positive gastric cancer through a circadian pathway involving PER1 and PPARγ. Metformin disrupts this pathway by inhibiting HK2 activity and promoting the degradation of PER1. Consequently, the combination of metformin with ZT6 trastuzumab has exhibited promising therapeutic potential against trastuzumab‐resistant gastric cancer.

##### 3.1.1.2 Enolase

Enolase, also known as phosphopyruvate hydratase, converts 2phosphoDglycerate to phosphoenolpyruvate (PEP)—a critical step in glycolysis that fuels ATP production and supports cancer cell proliferation and metastasis. In gastric cancer, elevated ENO1 expression is significantly associated with Lauren classification, lymph node metastasis, and TNM stage. Sun et al. [29] demonstrated that suppressing ENO1 not only reduces the growth and spread of gastric cancer cells but also reverses the epithelial–mesenchymal transition (EMT). Moreover, ENO1 regulates the AKT signaling pathway, which is essential for promoting gastric cancer cell proliferation and migration. In addition, Qian et al. [30] reported that in cisplatin‐resistant cells, increased glycolytic activity is linked to reduced miR22 expression, leading to higher ENO1 levels. Therefore, modulating ENO1 and miR22 expression may restore cisplatin sensitivity, offering a promising therapeutic strategy.

##### 3.1.1.3 Pyruvate Kinase M2

Pyruvate kinase M2 (PKM2) catalyzes the final step of glycolysis, converting PEP into pyruvate and producing ATP. Mammalian cells express four isoenzymes of pyruvate kinase—M1, M2, L, and R—each with specific expression patterns. Importantly, PKM2 is uniquely present in cancer cells, suggesting its potential role in tumor development.

Wang et al. [31] investigated the role of PKM2 in various gastric cancer cell lines with differing levels of differentiation. In well‐differentiated BGC823 and SGC7901 cells, which exhibit high expression of E‐cadherin, a reduction in PKM2 levels resulted in decreased E‐cadherin expression, thereby enhancing EGF/EGFR signaling and promoting invasion and metastasis. Conversely, in undifferentiated AGS cells that lack E‐cadherin, inhibition of PKM2 led to reduced migration and invasion. These contrasting outcomes suggest that the presence of E‐cadherin significantly influences the role of PKM2 in the progression of gastric cancer.

Further elucidating its mechanisms, Kwon et al. [32] demonstrated that PKM2 stabilizes the p65 protein, thereby enhancing its binding to the Bcl‐xL gene promoter and increasing Bcl‐xL transcription, which promotes gastric tumor growth. Additionally, Zieker et al. [33] reported that PKM2 acts as an upstream regulator of the PI3K/AKT signaling pathway; inhibition of PKM2 resulted in diminished PI3K‐Akt–mTOR activity, activation of autophagy, and a reduction in tumor migration. Moreover, the RNA‐binding protein PUM1 elevates DEPTOR levels, which inhibit mTORC1. This inhibition mitigates negative feedback on PI3K, consequently activating the PI3K‐Akt–mTOR pathway and enhancing glycolysis [34]. Furthermore, Zhao et al. [35] illustrated that circATP2B1 promotes glycolysis and cell proliferation in gastric cancer by sequestering miR‐326‐3p and miR‐330‐5p, thus reducing their inhibitory effects on PKM2.

In summary, these findings underscore the multifaceted roles of PKM2 in gastric cancer progression and highlight its potential as a therapeutic target.

##### 3.1.1.4 Lactate Dehydrogenase

LDH is an enzyme that converts pyruvate to lactate, playing a key role in tumor metabolism. In gastric cancer, LDH levels are significantly elevated, thereby increasing glycolytic activity. This alteration in enzyme expression is modulated by various transcription factors; for example, FOXO1 enhances the expression of the LDHA M subtype—specifically LDHA31—while decreased LDHA levels, influenced by OCT4, are associated with a favorable prognosis [36]. Overall, changes in LDH expression not only affect tumor metabolism but may also serve as valuable biomarkers for prognosis.

##### 3.1.1.5 Glucose Transporters

Glucose transporters (GLUTs) are essential for the uptake of glucose and other sugars in tumor cells. In gastric cancer, studies have highlighted distinct roles for different GLUT isoforms. For example, Yao et al. [37] reported that GLUT3 is upregulated in human gastric cancer and is associated with poor clinical outcomes. Mechanistically, elevated GLUT3 expression not only enhances glucose uptake and energy metabolism but also modulates glycolysis, activates the STAT3 pathway, promotes macrophage infiltration, and induces M2 polarization within the tumor microenvironment. Moreover, Yang et al. [38] demonstrated that increased GLUT3 levels further enhance glucose oxidation and lactate accumulation, leading to histone lactylation and facilitating EMT, which contributes to invasion and metastasis. In contrast, Ding et al. [39] found that miR‐148B suppresses GLUT1 expression, resulting in reduced glycolysis, decreased cell growth, and diminished invasiveness—suggesting that GLUT1 may serve as a potential biomarker and therapeutic target. Overall, these findings underscore the pivotal role of GLUT isoforms in tumor metabolism and gastric cancer progression.

##### 3.1.1.6 Hypoxia‐Inducible Factor 1‐Alpha

Activation of HIF1‐α plays a central role in regulating aerobic glycolysis by upregulating glycolytic genes and enhancing the pentose phosphate pathway. Zhang et al. [40] demonstrated that AAED1 activates ERK1/2 and AKT1, which in turn elevate HIF1‐α levels and boost glycolysis. Moreover, HIF1‐α modulates pyruvate dehydrogenase kinase (PDK), redirecting pyruvate toward lactate production [41]. Supporting this mechanism, Hur et al. [42] reported that high PDK‐1 expression correlates with tumor progression. In addition, the PDK‐1 inhibitor dichloroacetic acid (DCA) decreases lactate production and increases sensitivity to 5‐fluorouracil (5‐FU), highlighting its potential as a therapeutic agent. Similarly, Yang et al. [43] found that the acetyltransferase NAT10 influences SEPT9 mRNA levels, thereby activating the HIF1‐α pathway and altering glucose metabolism. Collectively, this interconnected feedback loop reinforces glycolysis and promotes tumor survival under hypoxic conditions.

##### 3.1.1.7 Phosphoglycerate Kinase 1

Phosphoglycerate kinase 1 (PGK1) plays a crucial role in ATP production through glycolysis and is significantly overexpressed in gastric cancer tissues [33]. This elevated PGK1 expression is not only linked to increased invasiveness but also closely associated with cancer progression. Additionally, PGK1 interacts with key proteins, such as CXCR4, modulating their expression levels. Understanding these interactions could provide valuable insights into the mechanisms of metastasis and identify potential therapeutic targets.

#### 3.1.2 Tricarboxylic Acid Cycle

The citric acid cycle, also known as the tricarboxylic acid (TCA) cycle or Krebs cycle, plays a crucial role in cellular growth and development by generating the energy and precursor molecules needed for biosynthesis. This cycle primarily occurs in the mitochondria, where it produces NADH and FADH_2_. These molecules are essential for powering the mitochondrial electron transport chain (ETC), which in turn synthesizes ATP. Moreover, the TCA cycle marks the final step in the breakdown of sugars, fats, and amino acids, producing vital intermediates required for cellular synthesis [44, 45]. Several enzymes involved in the TCA cycle—such as succinate dehydrogenase (SDH), fumarate hydratase (FH), and isocitrate dehydrogenase (IDH)—have been implicated in cancer development [45]. Among these, SDH is particularly linked to gastric cancer. This enzyme converts succinate to fumarate while reducing ubiquinone to ubiquinol within the ETC. The SDH enzyme consists of four subunits: SDHA, SDHB, SDHC, and SDHD, which are embedded in the inner mitochondrial membrane. Its activity is regulated by the nuclear genes SDHAF1 and SDHAF2 [44, 45]. By reducing succinate levels and inhibiting HIF‐1α, SDH helps suppress tumor growth. However, mutations in the SDH gene can result in tumor formation and genomic instability [45].

Numerous studies have highlighted significant changes in TCA cycle metabolites during metabolic reprogramming in cancer, underscoring their role in the progression of gastric cancer. For instance, Kim et al. [46] developed a method that combines quaternary ammonium pairing with freeze‐drying to analyze short‐chain fatty acids (SCFAs) and TCA intermediates in human plasma via GC–MS/MS. They found that gastric cancer patients exhibited significantly higher levels of SCFAs, including acetic, propionic, and butyric acids, alongside lower levels of citric and malic acids in their plasma. Similarly, Chen et al. [47] utilized mass spectrometry coupled with capillary electrophoresis (MRB‐CE–MS) to study urinary metabolomics in gastric cancer patients. They reported increased lactic acid and decreased citric, malic, and succinic acids, suggesting enhanced glycolysis and reduced TCA cycle activity. Gu et al. [48] observed increased succinate levels in gastric cancer rat tissues. Additionally, Liang et al. [49], through LC–MS analysis, found higher levels of citric and malic acids and lower levels of succinic acid in the urine of gastric cancer patients. Finally, Song et al. [50] reported elevated levels of metabolites associated with aerobic glycolysis, particularly fumaric and α‐ketoglutaric acids, in tumor tissues via GC/MS.

This comprehensive evaluation of TCA cycle intermediates via various methodologies underscores their potential to serve as biomarkers for disease progression and as targets for therapeutic intervention in gastric cancer.

#### 3.1.3 Pentose Phosphate Pathway

The pentose phosphate pathway (PPP), a branch of glycolysis, oxidizes glucose to produce ribose 5‐phosphate and nicotinamide adenine dinucleotide phosphate (NADPH). Ribose 5‐phosphate is essential for nucleotide synthesis, while NADPH plays a critical role in maintaining redox balance and supporting the biosynthesis of compounds such as tetrahydrofolate, deoxyribonucleotides, proline, fatty acids, and cholesterol. Notably, NADPH facilitates fatty acid synthesis and neutralizes reactive oxygen species (ROS), enabling glycolytic cancer cells to meet their metabolic needs and manage oxidative stress [51, 52]. In addition, Tao et al. [53] demonstrated that Rev‐erbα—a nuclear receptor involved in regulating circadian rhythms, inflammation, and lipid metabolism—suppresses both glycolysis and the PPP by binding to the promoters of the PFKFB3 and G6PD genes in human gastric cancer cells, thereby limiting their proliferation. Gastric cancer samples with low levels of Rev‐erbα show increased PPP and glycolytic activity compared to those with relatively high Rev‐erbα levels, which may contribute to cancer progression. Therefore, targeting the metabolic changes regulated by Rev‐erbα could provide new therapeutic strategies for gastric cancer.

In conclusion, carbohydrate metabolism is crucial for supplying energy to gastric cancer cells. Analyzing the complexities of carbohydrate metabolism in these cells provides valuable insights into gastric cancer progression and helps identify potential biomarkers for targeting metabolic pathways. Continued exploration of carbohydrate metabolism in gastric cancer not only enhances our understanding of the disease but also opens opportunities for the development of novel therapeutic strategies in clinical settings.

## 4 Amino Acid Metabolism and Gastric Cancer

In mammalian cells, amino acids primarily function as building blocks for proteins. Beyond protein synthesis, they play key roles in energy production, nucleotide synthesis, and the maintenance of cellular redox balance. Cancer cells, compared to normal cells, have a significantly higher demand for amino acids to support their rapid growth, as they preferentially use amino acids over traditional energy pathways [54, 55]. Elevated amino acid levels are often associated with increased tumor cell proliferation. Importantly, amino acid metabolism is closely linked not only to tumor development but also to clinical outcomes and treatment responses [55]. To meet the demands of malignant growth, tumor cells adopt various strategies to acquire essential amino acids. For example, they can extract proline from the extracellular matrix to generate ATP, which further promotes tumor growth and spread [54]. Understanding these amino acid metabolic pathways is crucial for unraveling the complexity of tumor biology and offers potential avenues for developing new therapeutic strategies.

**FIGURE 2:**
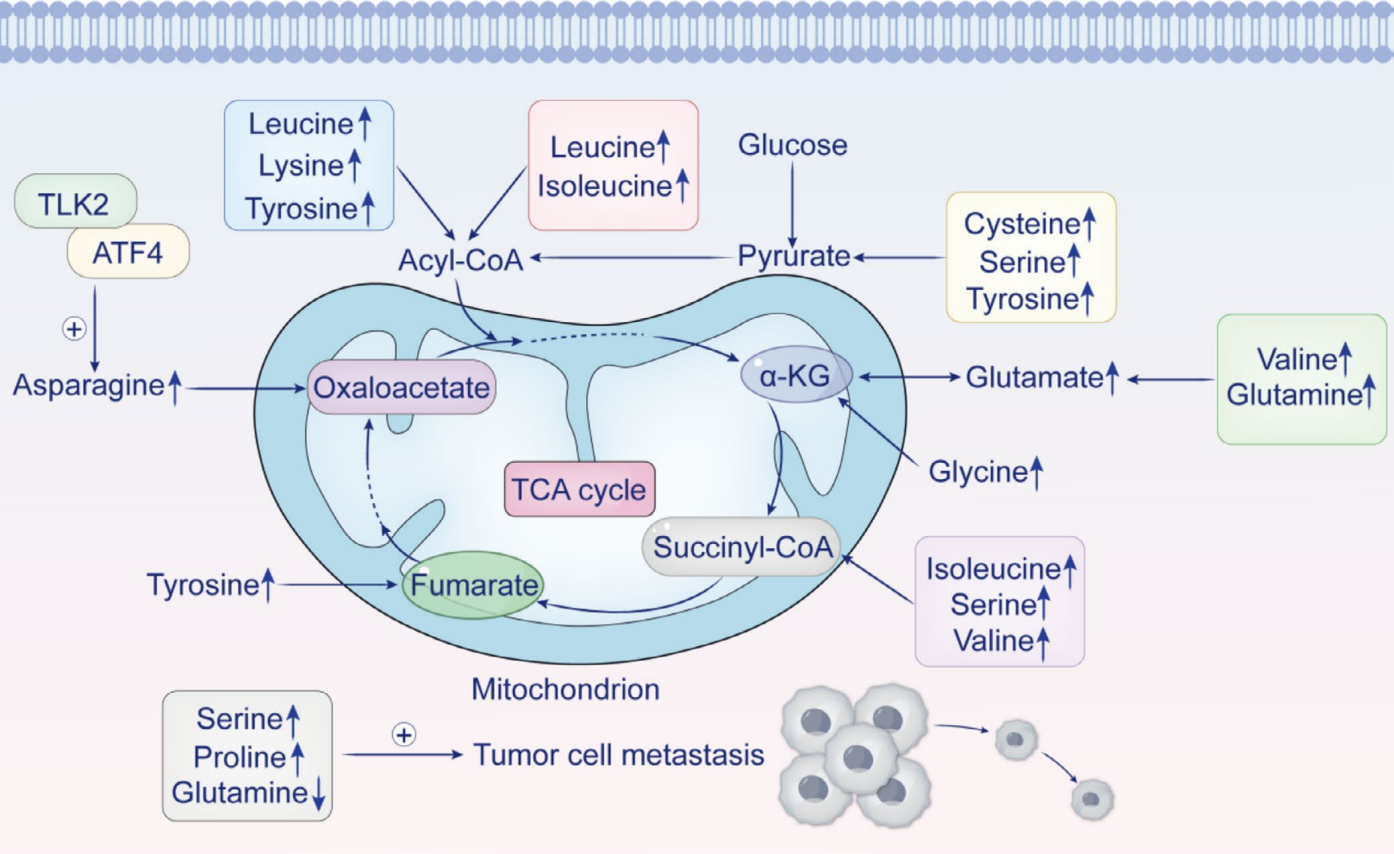
Mechanisms of amino acid metabolism in gastric cancer. Amino acid metabolism in gastric cancer cells is a critical area of study. The figure illustrates key pathways involving amino acids such as leucine, isoleucine, serine, proline, and glutamine within the tricarboxylic acid (TCA) cycle. Enzymes like TLK2 and ATF4 play significant roles in regulating the stability and synthesis of asparagine synthetase (ASNS), thereby facilitating metabolic reprogramming and promoting the metastasis of gastric tumors. Furthermore, the roles of serine, proline, and glutamine in promoting tumor cell metastasis are also illustrated. ATF4, activating transcription factor 4; TLK2, Tousled‐like kinase 2.

The mechanisms of amino acid metabolism in gastric cancer are shown in Figure 2.

Recent advancements in metabolomics are pivotal for investigating metabolic anomalies and identifying potential biomarkers across various cancers. For instance, Yu et al. [56] reported elevated levels of glutamate, cysteine, and glycine specifically in gastric cancer, highlighting their significance in this disease. Utilizing GC–MS analysis, Chen et al. [57] elucidated the critical roles of proline and serine in the progression of gastric cancer. They identified proline as the most abundant metabolic product within metastatic tissues and noted a significant reduction in glutamine levels, suggesting that these metabolites may serve as promising biomarkers for managing metastatic gastric cancer.

Additionally, Wang et al. [58], employing ^1^H NMR spectroscopy alongside multivariate statistical techniques, observed increased concentrations of glutamine, glutamate, and serine in gastric cancer tissues compared to noncancerous counterparts; they also reported heightened levels of isoleucine, leucine, valine, lysine, and tyrosine. Furthermore, Wang et al. [59] discovered an upregulation of activating transcription factor 4 (ATF4) expression in both gastric cancer cells and tissues. This finding indicates that ATF4 plays a regulatory role in asparagine metabolism while influencing the autophagy pathway through mTORC1 signaling.

In summary, these findings highlight the potential of metabolomic analysis to identify critical metabolic biomarkers and pathways associated with gastric cancer—insights that could ultimately improve diagnostic accuracy and support the development of targeted therapeutic strategies.

TLK2 is considered an oncogene that promotes gastric cancer progression by altering amino acid metabolism. It enhances the stability of the asparagine synthetase (ASNS) protein by inhibiting its ubiquitination. In addition, TLK2 interacts with the transcription factor ATF4, which regulates ASNS protein levels and increases its transcriptional activity [60]. Despite these insights, research on amino acid metabolic changes in gastric cancer remains limited. Therefore, exploring these specific alterations through metabolomics could deepen our understanding of gastric cancer biology and uncover new therapeutic targets, offering more effective treatment strategies for patients.

The relationship between glutamine metabolism and gastric cancer is shown in Table 2.

**TABLE 2:** Metabolism of glutamine and its association with gastric cancer.

Study year	Mechanism	Focus	Key insights	Reference
2023	CDC42/GLS1	M2 macrophage polarization; increased glutamine metabolism; tumor growth	Cancer cells use CDC42 to release GLS1‐containing vesicles, boosting glutamine metabolism, and promoting M2 macrophage polarization. This process aids macrophage‐mediated angiogenesis and contributes to trastuzumab resistance in HER2‐positive gastric cancer	[61]
2022	CircAKT3/miR‐515‐5p	Cell proliferation and survival; glutamine metabolism	CircAKT3 promotes gastric cancer cell growth and survival by upregulating SLC1A5, enhancing glutamine metabolism	[62]
2023	RACK1/AKT/mTOR	Glutamine dependence; AKT/mTOR/ASCT2 pathway	Loss of RACK1 promotes glutamine addiction through the AKT/mTOR/ASCT2 pathway, fueling gastric cancer growth	[63]
2023	NR_033928/GLS Stabilization	Reprogrammed glutamine metabolism	The m5C‐methylated lncRNA NR_033928 stabilizes GLS mRNA, reprogramming glutamine metabolism, and promoting gastric cancer cell proliferation	[64]
2022	RUNX3/CircDYRK1A	Glutamine Metabolism	RUNX3 inhibits glutamine metabolism by upregulating FBXO4 via microRNA‐889‐3p, affecting the malignant traits of gastric cancer	[65]

## 5 Lipid Metabolism and Gastric Cancer

Lipids, including fatty acids, triglycerides, phospholipids, cholesterol, cholesterol esters, and sphingolipids, are essential components of cell membranes [66]. During cancer progression, lipids not only provide energy and maintain membrane integrity but also act as signaling molecules, such as PGE2, S1P, and LPA. Additionally, lipids influence epigenetic modifications through the acylation of key molecules [67], thereby regulating cancer cell behavior. Abnormal lipid metabolism is a hallmark of cancer cells, enabling them to generate energy and store fats by increasing de novo lipogenesis, fatty acid uptake, and FAO [68]. Cancer cells also utilize lipids to acquire fatty acids, supporting lipid synthesis and assimilation. This highlights the crucial role of fatty acids in tumor growth, progression, and metastasis [69]. Understanding lipid metabolism in cancer not only reveals the complexity of tumor biology but also provides valuable insights for developing new therapeutic strategies.

### 5.1 Ferroptosis‐Related Lipid Metabolism in Gastric Cancer

Ferroptosis is an iron‐dependent form of regulated cell death triggered by lipid peroxidation [70, 71]. Zhang et al. [70] reported that exosomal miR‐522, released by CAFs, inhibits ferroptosis in gastric cancer cells by targeting ALOX15. This prevents the accumulation of lipid‐derived reactive oxygen species (lipid‐ROS), thereby promoting tumor progression. In contrast, Lee et al. [71] found that mesenchymal‐type gastric cancer cells are more sensitive to ferroptosis due to the upregulation of ELOVL5 and FADS1. However, in intestinal‐type gastric cancer cells, DNA methylation silences these enzymes, leading to ferroptosis resistance. Additionally, lipidomic analysis and isotope tracing revealed that intestinal‐type gastric cancers cannot convert linoleic acid into arachidonic acid (AA) and adrenic acid (AdA). However, supplementing with AA restores their sensitivity to ferroptosis. These findings underscore the significant role of the polyunsaturated fatty acid (PUFA) synthesis pathway in regulating ferroptosis, suggesting its potential as a predictive marker for ferroptosis‐targeted therapies.

Yao et al. [72] reported that the reduced expression of FA2H in gastric cancer tissues, compared to adjacent normal tissues, is associated with clinical outcomes, highlighting its potential role in tumor proliferation and chemotherapy resistance. This resistance is linked to the inhibition of AMPK, activation of the mTOR/S6K1/Gli1 pathway, and the resulting increase in Gli1 levels within tumors. Notably, increasing FA2H levels or administering (R)‐2‐hydroxy fatty acid ((R)‐2‐OHFA) significantly lowers Gli1 levels in gastric tumors, which in turn enhances sensitivity to cisplatin and reduces chemotherapy‐related weight loss in mice. Taken together, these findings underscore the crucial role of FA2H in regulating Hedgehog signaling and gastric tumor progression. Moreover, they suggest that (R)‐2‐hydroxy fatty acids could serve as nontoxic agents to improve the effectiveness of chemotherapy. Consequently, modulating ferroptosis and related pathways presents a promising therapeutic strategy for gastric cancer.

### 5.2 Lipid Metabolism Related to Metastasis in Gastric Cancer

The mechanisms underlying peritoneal metastasis (PM) in cancer remain incompletely understood. However, Fang et al. [73] emphasized the critical role of laminin subunit gamma 1 (LAMC1) in establishing a microenvironment conducive to PM. Specifically, LAMC1 facilitates the maturation of preperitoneal adipocytes, which release free fatty acids (FFAs), thereby significantly advancing the progression of metastatic gastric cancer. In a related study, He et al. [74] reported that LINC00924, a long noncoding RNA, is closely associated with PM in gastric cancer. Elevated expression of LINC00924 correlates with the extent of PM, and further investigations revealed that LINC00924 enhances the interaction between hnRNPC and Mnk2 pre‐mRNA exon 14a. This interaction inhibits Mnk2a splicing, consequently affecting the p38 MAPK/PPARα signaling pathway. Ultimately, this regulatory cascade enhances FAO and fatty acid uptake in gastric cancer cells, thereby promoting tumor progression.

In a clinical study, Jia et al. [75] reported that RPRD1B gene levels are significantly elevated in the metastatic lymph nodes of gastric cancer patients and are associated with poor prognosis. Further research shows that RPRD1B overexpression enhances the transcription of c‐Jun and c‐Fos, which activates the c‐Jun/c‐Fos/SREBP1 pathway, promoting fatty acid uptake and biosynthesis and driving metastasis. Additionally, elevated NEAT1 expression was observed in cells with increased c‐Jun and c‐Fos levels due to RPRD1B overexpression. NEAT1 plays a key role in m6A modification, which enhances RPRD1B mRNA stability. These findings suggest that m6A‐mediated stabilization of RPRD1B may initiate mechanisms that contribute to gastric cancer metastasis to lymph nodes. In conclusion, these interconnected pathways underscore the complexity of gastric cancer metastasis, particularly with respect to peritoneal and lymph node involvement. Insights from these studies reveal potential therapeutic targets that could disrupt metastasis and improve clinical outcomes for patients with advanced gastric cancer.

### 5.3 Carnitine Palmitoyltransferase 1A

Carnitine palmitoyltransferase 1A (CPT1A), an enzyme critical for FAO, is found to be overexpressed in various cancers. Zhu et al. [76] reported that a small protein derived from lncAKR1C2, secreted by gastric cancer cells, enhances FAO and ATP production by modulating CPT1A expression through the Yes‐associated protein (YAP). This small protein interacts with YAP, leading to a reduction in its phosphorylation and promoting its translocation into the nucleus, which subsequently increases CPT1A levels and augments both FAO and ATP synthesis. Furthermore, Wu et al. [77] discovered that the lncRNA HCP5, induced by mesenchymal stem cells, activates CPT1 transcription via the miR‐3619‐5p/AMPK/PGC1α/CEBPB pathway. This activation results in increased FAO within gastric cancer cells while also enhancing stemness and conferring chemotherapy resistance. These findings suggest that targeting HCP5 may improve the efficacy of chemotherapy in patients diagnosed with gastric cancer.

Additionally, NPRA, a receptor belonging to the guanylyl cyclase family, plays a crucial role in maintaining water and sodium balance. Research [78] has indicated that tumors overexpressing NPRA exhibit elevated levels of peroxisome proliferator‐activated receptor alpha (PPARα). Both in vitro and in vivo studies have confirmed that NPRA interacts with PPARα, thereby preventing its degradation. This stabilization enables PPARα to activate CPT1B, which promotes FAO. In summary, the regulation of FAO through the modulation of CPT1A and CPT1B by factors such as lncAKR1C2, HCP5, and NPRA underscores the complexity of metabolic reprogramming in cancer. This highlights potential therapeutic targets for disrupting FAO pathways and improving treatment outcomes for gastric cancer.

### 5.4 Signal Transducer and Activator of Transcription 5A

The nuclear transcription factor STAT5A is activated by cytokines or growth factor receptors and plays a crucial role in immune responses, inflammation, and cancer progression. Dong et al. [79] reported that STAT5A upregulation in gastric cancer is associated with signaling pathways involved in fatty acid metabolism. Specifically, STAT5A interacts with the promoter region of FABP5, regulating the expression of fatty acid‐binding protein 5 in MKN28 and AGS cells. Overexpression of FABP5 significantly enhances the tumorigenic potential of gastric cancer cells in animal models, leading to increased intracellular fatty acid levels and promoting cancer progression. In summary, these findings suggest that the interaction between STAT5A and FABP5 is crucial for promoting fatty acid metabolism and tumor development in gastric cancer, highlighting it as a potential therapeutic target.

### 5.5 Stearoyl‐CoA Desaturase

Increased expression of stearoyl‐CoA desaturase (SCD) has been associated with the development of gastrointestinal cancers. Won et al. [80] reported a metabolic shift from glycolysis to fatty acid metabolism, wherein altered fatty acid desaturation promotes dysplastic cell proliferation in a mouse model featuring Kras activation in gastric chief cells. Inhibition of SCD, which is upregulated during carcinogenesis, selectively induced apoptosis in dysplastic cells across both mouse and human models, underscoring its potential as a therapeutic target. These findings suggest that fatty acid metabolism supports the high energy demands of dysplastic cells during gastric cancer progression.

Disruptions in lipid metabolism, as well as changes in carbohydrate and amino acid metabolism, play a crucial role in cancer progression. Alterations in lipid pathways, in particular, are considered hallmarks of aggressive tumors, offering potential targets for novel anticancer therapies. By focusing on these disrupted pathways, researchers can develop innovative strategies to combat cancer and improve patient outcomes. Thus, understanding and modulating lipid metabolism in cancer cells is vital for advancing treatment options and achieving clinical success.

## 6 (Mitochondrial) One‐Carbon Metabolism and Gastric Cancer

Recent research has highlighted the critical role of mitochondrial one‐carbon metabolism in cancer development. This metabolic network links the cytoplasm and mitochondria through pathways such as the folate cycle, methionine cycle, and transsulfuration pathway. These biochemical reactions provide methyl groups essential for various metabolic processes, including the synthesis of thymine, methionine, serine, glycine, and purines [81, 82]. Furthermore, one‐carbon metabolism facilitates cancer cell proliferation and growth by supplying precursors for nucleotides and amino acids [82].

Yoon et al. [83] identified a chemoresistant subtype of gastric cancer termed SEM‐type. This subtype is characterized by elevated levels of glutaminase (GLS) and distinct metabolic flexibility. The survival of SEM‐type cells is driven by transcription factors ATF4 and CEBPB. These cells enhance their viability by upregulating a mitochondrial one‐carbon metabolism pathway mediated by 3‐phosphoglycerate dehydrogenase (PHGDH), which generates NADPH while scavenging reactive oxygen species. This study suggests that targeting both the GLS pathway and the PHGDH pathway may represent a promising therapeutic strategy for patients with chemotherapy‐resistant gastric cancer, as validated in patient‐derived organoids.

## 7 Metabolomics in the Diagnosis of Gastric Cancer

Metabolomics is an essential tool for studying metabolic reprogramming and is a rapidly growing field that builds upon genomics and proteomics. This field primarily focuses on metabolites—small molecules typically weighing less than 1000 Da—to assess the types, quantities, and trends of metabolic byproducts in response to changes in gene expression, enzyme function, metabolic pathways, or environmental factors [84]. Key techniques used in metabolomics include nuclear magnetic resonance (NMR), liquid chromatography–mass spectrometry (LC–MS), ultra‐performance liquid chromatography–mass spectrometry (UPLC/MS), gas chromatography–mass spectrometry (GC–MS), and capillary electrophoresis–mass spectrometry (CE–MS) [85, 86, 87].

Recent studies have demonstrated the potential of metabolomics in improving gastric cancer diagnosis and prognosis. For instance, Chen et al. [88] employed a targeted metabolomics approach using LC–MS to analyze plasma samples from 702 gastric cancer patients and noncancer controls. Their analysis led to the development of a diagnostic model based on 10 metabolites, which outperformed traditional protein marker methods. Additionally, they established a prognostic model to stratify patients according to risk, thus enhancing precision medicine for gastric cancer patients. Similarly, Sergio et al. [89] collected plasma samples from patients with various gastric conditions and analyzed 208 metabolites using a targeted LC–MS method. Their study revealed significant alterations in the metabolic profiles of gastric cancer patients, particularly within the tryptophan and nitrogen metabolism pathways. These studies underscore the necessity for further metabolomics research using absolute quantification to achieve the precision, accuracy, and reproducibility required for reliable diagnostics and biomarker validation.

In another study, Yuan et al. [90] performed metabolomic profiling on 112 gastric cancer patients and 112 healthy controls, reporting relationships between alanine, aspartate, and glutamate metabolism, glycolysis/gluconeogenesis, and HER2 levels in gastric cancer. These findings highlight the potential of metabolomics in identifying key metabolic pathways and biomarkers relevant to cancer research.

The emergence of mass spectrometry imaging (MSI) technology provides a robust method for investigating the spatial distribution of metabolites and lipids within tumor tissues. Spatial metabolomics (SM) and spatial lipidomics (SL), which leverage MSI techniques, facilitate the exploration of metabolic biomarkers linked to tumor initiation, progression, and metastasis in their native context [91, 92, 93, 94, 95, 96]. This approach significantly enhances our comprehension of the metabolic frameworks present within tumors and their microenvironments.

For instance, Sun et al. [97] proposed a comprehensive strategy that integrates SL and metabolomics with spatial transcriptomics through microarrays. This innovative methodology enables the visualization of metabolic variability and cellular interactions within individual gastric cancer samples, thereby allowing for an examination of spatial differences in the tumor microenvironment while elucidating connections among various metabolic pathways. Similarly, Wang et al. [98] performed a SM analysis on 362 gastric cancer patients utilizing high‐resolution imaging mass spectrometry. Their study identified distinct tumor‐specific and matrix‐specific subtypes characterized by unique tissue metabolic signatures. Notably, patients classified as T1 (HER2+MIB+CD3+) exhibited elevated nucleotide levels, enhanced DNA metabolism, and more favorable prognoses in comparison with those categorized as T2 or T3.

In conclusion, tissue‐based SM for subtyping offers vital insights that can improve molecular classification systems for gastric cancer. By understanding the metabolic differences among subtypes and their links to molecular features, we can tailor treatment approaches for more effective and personalized therapies. Gastric cancer development involves metabolic disturbances in glucose, amino acid, and lipid metabolism. Thus, metabolomics research has significant potential to identify new biomarkers for diagnosis, predict treatment responses, and elucidate the pathogenesis of gastric cancer. Investigating these metabolic changes enhances our understanding of disease mechanisms and paves the way for innovative diagnostic and therapeutic strategies that ultimately improve patient outcomes.

A summary of the researchers, their findings, and the analytical technologies used is shown in Table 3.

**TABLE 3:** Researchers and their related findings and analysis technology.

Authors	Key discoveries	Techniques
Chen [88]	Developed a 10‐metabolite diagnostic model for gastric cancer, exceeding traditional protein markers Created a prognostic model to inform personalized treatment and improve precision medicine	LC–MS
Sergio [89]	Gastric cancer patients showed notable changes in their metabolic profiles Pathway analysis indicated alterations in tryptophan and nitrogen metabolism	LC–MS
Yuan [90]	Identified connections between metabolites such as alanine and aspartic acid and HER2 levels in gastric cancer patients via metabolomics analysis	Unspecified
Sun [97]	Employed an integrated approach (spatial lipidomics, metabolomics, and transcriptomics) to visualize metabolic diversity and cellular interactions in gastric cancer	Microarray and mass spectrometry imaging
Wang [98]	Identified subtypes specific to tumor and stroma in gastric cancer	High‐resolution mass spectrometry imaging

## 8 Metabolomics in Research and Medicine

Drug development relies heavily on the detection and measurement of target diseases, with the extent of disease typically assessed through biomarkers. Metabolomics methods have proven effective in identifying biomarkers across a wide array of biological samples, correlating with over 400 distinct disease conditions [99]. Consequently, metabolomics serves as a crucial tool for discovering universal biomarkers applicable to disease diagnosis and evaluating drug efficacy.

In a prospective study [100], an analysis of serum metabolic profiles from patients diagnosed with Alzheimer's disease revealed significant alterations in various metabolic biomarker features, including lipid metabolites, sphingosines, sphingomyelin, and sterols. These findings highlight the role of oxidative stress and disrupted lipid metabolism in the progression of Alzheimer's disease while also offering predictive insights into its advancement.

Another investigation [99] examined the effects of chloroethylnitrosourea on murine tumor models through the application of metabolomics techniques. The findings revealed that glucose, glutamine, and aspartic acid were significantly accumulated in all tumors following the administration of chloroethylnitrosourea; this accumulation was associated with inhibition within the de novo synthesis pathway for nucleotides. Consequently, these metabolites may serve as potential biomarkers for evaluating the efficacy of this anticancer agent.

Metabolomics can significantly improve the identification of drug targets for complex diseases. Novel cancer chemotherapy targets have been discovered through metabolomic analyses, including N‐methylglycine in prostate cancer and 2‐hydroxyglutaric acid in acute myeloid leukemia [101]. Additionally, elevated glutaminolysis has been observed in lung squamous cell carcinoma cells. Momcilovic et al. [102] employed isotope tracing and flux analysis to specifically target glutamine metabolism, effectively addressing treatment resistance seen in vivo.

Drug uptake, distribution, metabolism, excretion, and toxicology (ADMET) are critical yet time‐consuming processes in drug development. Traditional research methods tend to be invasive, costly, error‐prone, and slow. In contrast, metabolomics offers a simpler, less invasive, and faster detection method. Lane et al. [103] employed the Stable Isotope Resolved Metabolomics (SIRM) technique to investigate the effects of drugs on human lung cancer cell metabolism in both in vitro and in vivo models. This approach provides more detailed insights into molecular mechanisms compared to traditional methods.

There are significant individual differences in drug response. Metabolomics can enhance treatment plans and advance personalized medicine. Caridad Diaz et al. [104] proposed a nontargeted metabolomics approach using liquid chromatography–high‐resolution mass spectrometry (LC‐HRMS) to detect plasma molecular changes across breast cancer subtypes under the same neoadjuvant chemotherapy (NACT) regimen, aiming to identify potential predictors of response. Notable metabolite differences were observed between responders and nonresponders in the triple‐negative TN subtype, potentially linked to treatment efficacy. Glycine chenodeoxycholic acid (GDCA) and glycine porcine deoxycholic acid (GHCA) emerged as potential biomarkers for TN isoform response. This study provides insights into personalized breast cancer therapy and emphasizes metabolomics' role in early detection of chemotherapy resistance.

Yang et al. [105] utilized UHPLC‐QTOF/MS analysis to predict CRC patients' responses to NACT. Nine metabolites showed a strong correlation with NACT sensitivity, offering higher predictive accuracy than standard clinical biomarkers. These findings present a fresh perspective on personalized CRC treatment with significant clinical implications.

Zhang et al. [106] discovered five differentially expressed metabolites in esophageal squamous cell carcinoma (ESCC) patients who had not undergone neoadjuvant chemoradiotherapy (nCRT) through GC‐TOF/MS nontargeted metabolomics. These metabolic changes may indicate ESCC patients' sensitivity to nCRT, paving the way for identifying biomarker candidates for predicting nCRT response.

## 9 Conclusion and Outlook

Gastric cancer is a significant global health issue, being the fourth leading cause of cancer‐related deaths. Early detection is often lacking, resulting in diagnoses at advanced stages and poor prognoses. Patients with advanced or metastatic gastric cancer have much lower 5‐year survival rates compared to those with other cancers.

To survive and adapt under unfavorable conditions, cancer cells alter their metabolism to increase energy acquisition. These changes impact the metabolism of carbohydrates, lipids, and amino acids, leading to variations in small‐molecule metabolites. Such alterations in metabolites hold promise as potential biomarkers for early detection and prognosis. Metabolomics, a noninvasive technique for identifying and quantifying small molecules in cancerous tissues, has emerged as a powerful tool. This approach not only identifies biomarkers for early gastric cancer but also enhances our understanding of the molecular pathways that drive its progression.

Despite the potential of metabolomics, current research is limited and inconsistent, hindering its clinical integration. These challenges arise from methodological differences and technological constraints. Identifying metabolites with complex structures or low concentrations remains difficult, and existing databases are inadequate for accurate identification. The wide dynamic range of the metabolome complicates the detection of low‐abundance metabolites. Variations in sample handling, environmental conditions, and lifestyle factors can significantly influence study outcomes, thereby affecting their accuracy and reliability.

Future studies should aim to integrate diverse research approaches, identify key metabolic targets, promote technological advancements, and strengthen ties to clinical practice. This strategy could revolutionize gastric cancer diagnosis and treatment, ultimately improving patient outcomes and reducing mortality rates.

## Author Contributions

**Yu Rong:** writing – original draft (lead), writing – review and editing (equal). **Yuanyin Teng:** writing – review and editing (equal). **Xiaoying Zhou:** conceptualization (supporting), supervision (lead).

## Conflicts of Interest

The authors declare no conflicts of interest.

## Data Availability

The authors have nothing to report.
